# Avian Influenza Viruses Infect Primary Human Bronchial Epithelial Cells Unconstrained by Sialic Acid α2,3 Residues

**DOI:** 10.1371/journal.pone.0021183

**Published:** 2011-06-23

**Authors:** Christine M. Oshansky, Jennifer A. Pickens, Konrad C. Bradley, Les P. Jones, Geraldine M. Saavedra-Ebner, James P. Barber, Jackelyn M. Crabtree, David A. Steinhauer, S. Mark Tompkins, Ralph A. Tripp

**Affiliations:** 1 Department of Infectious Diseases, College of Veterinary Medicine, University of Georgia, Athens, Georgia, United States of America; 2 Department of Microbiology and Immunology, Emory University School of Medicine, Atlanta, Georgia, United States of America; University of Hong Kong, Hong Kong

## Abstract

Avian influenza viruses (AIV) are an important emerging threat to public health. It is thought that sialic acid (sia) receptors are barriers in cross-species transmission where the binding preferences of AIV and human influenza viruses are sias α2,3 versus α2,6, respectively. In this study, we show that a normal fully differentiated, primary human bronchial epithelial cell model is readily infected by low pathogenic H5N1, H5N2 and H5N3 AIV, which primarily bind to sia α2,3 moieties, and replicate in these cells independent of specific sias on the cell surface. NHBE cells treated with neuraminidase prior to infection are infected by AIV despite removal of sia α2,3 moieties. Following AIV infection, higher levels of IP-10 and RANTES are secreted compared to human influenza virus infection, indicating differential chemokine expression patterns, a feature that may contribute to differences in disease pathogenesis between avian and human influenza virus infections in humans.

## Introduction

Influenza A viruses are important pathogens that present a significant threat to public health, causing an extensive economic burden particularly for avian influenza virus (AIV) infection of poultry. Influenza viruses are segmented, enveloped, negative-strand RNA viruses belonging to the *Orthomyxoviridae* family. They comprise a diverse array of subtypes due to their propensity to change antigenic profiles and are subtyped based on the antigenic properties of two surface glycoproteins, i.e. hemagglutinin (HA) and neuraminidase (NA). Seasonal epidemics cause more than 200,000 hospitalizations and more than 41,000 deaths each year in the United States alone [1]. Four novel influenza viruses caused pandemics in 1918, 1957, 1968, and most recently in 2009. The 1918 influenza pandemic was the most severe resulting in unusually high mortality among healthy young adults [2]. It remains unclear the precise features that contributed to the high rate of mortality due to infection with the 1918 influenza virus, but it has been shown that a single mutation in the PB1-F2 genome of 1918 influenza A viruses (also recognized for highly pathogenic H5N1 avian influenza) contributed to increased virulence [3], [4], [5]. Moreover, since 2003, there has been an increased incidence of highly pathogenic avian influenza (HPAI) virus outbreaks in poultry, and HPAI H5N1 has crossed species barriers to infect >500 humans resulting in nearly a 60% fatality rate (>300 deaths) as of April 2011 [6].

Influenza HA binds to host cell sialic acid residues (sias) coating the host cell surface [7] and mediates viral entry via its receptor binding domain. Human influenza viruses preferentially bind sia α2,6 linkages, while AIV preferentially bind sia α2,3 linkages that are highly expressed in the gastrointestinal tracts of aquatic birds [8], [9], [10], [11], [12], [13], [14], [15], thus it is thought that sialic acid residues are important barriers in cross-species transmission. Sias are nine-carbon monosaccharides found at the ends of glycan chains. Sias coat many host cell surfaces and secreted proteins [16], [17], [18], [19]. The most common sias found in mammals are N-acetylneuraminic acid (Neu5Ac) and N-glycolylneuraminic acid (Neu5Gc). Sias are transferred to terminal sugars of glycoproteins and glycolipids by sialyltransferases, and can be added to the galactose carbon-6 forming an α2,6 linkage or to galactose carbon-3 forming an α2,3 linkage [14], [16], [19]. The detection of α2,3 or α2,6 linkages can be determined by use of plant lectins that specifically bind to glycolipids and glycoproteins containing sia α2,6 or α2,3 configurations. A lectin from the seed of *Maackia amurensis* tree (MAA) is specific for sia α2,3, while a lectin obtained from the elderberry plant *Sambucus nigra* (SNA) is specific for sia α2,6 [20], [21]. Early experiments showed that SNA preferentially bound to the surface of ciliated tracheal epithelial cells indicating the presence of sia α2,6, and MAA bound goblet cells indicating the presence of sia α2,3 [22]. These studies suggested that ciliated cells, but not goblet cells, were a primary target for human H3 influenza infection and were subsequently confirmed by using a fluorescently-labeled H3 virus which primarily attached to ciliated cells [23]. However, later studies using differentiated human tracheal bronchial epithelial cells found that human influenza viruses infect non-ciliated cells expressing sia α2,6, and AIV infect ciliated cells expressing sia α2,3 [24]. More recent evidence suggests that H5N1 influenza can replicate within *ex vivo* human respiratory epithelial tissues, despite the lack of sia α2,3 staining [25]. Regardless of the predilection of AIV for sia α2,3, a H5N1 AIV (A/Hong Kong/156/1997) outbreak occurred in humans in Hong Kong in 1997 where all eight viral genes were of avian origin. The currently circulating H5N1 AIV strains primarily infect birds and fowl maintaining a sia α2,3 binding preference; however, AIV can acquire mutations changing their HA binding specificity from avian-like, α2,3, to human-like, α2,6 [8], [10], [26].

In these studies, we determined if low pathogenic H5N1, H5N2 and H5N3 AIV isolates of chicken or wild bird origin could infect and replicate in fully differentiated, normal human bronchial epithelial (NHBE) cells. We show that these viruses infect, replicate, and are released from NHBE cells independent of detectable sia α2,3 or α2,6 moieties present on the cell surface, and show that LPAI H5N1, H5N2 and H5N3 viruses induce higher IP-10 and RANTES responses early during infection compared to human H3N2 infection indicating differential chemokine expression patterns that may contribute to the unique aspects of disease pathogenesis between avian and human influenza virus infection.

## Materials and Methods

### Cells and viruses

Normal human bronchial epithelial (NHBE) cells (Lonza, Walkersville, MD) from a single 17 year old healthy male donor were expanded, cryopreserved, and cultured in an air-liquid interface system as previously described [27]. The cells from the same donor were used in all assays for assay consistency. The apical surface of the cells was exposed to a humidified 95% air/5% CO_2_ environment, and the basolateral medium was changed every two days.

The low pathogenic AIV (LPAI) strains A/Mute Swan/MI/06/451072-2/2006 (H5N1), A/chicken/Pennsylvania/13609/1993 (H5N2), and A/chicken/TX/167280-4/02 (H5N3) were kindly provided by Dr. David Suarez, USDA-Southeast Poultry Research Laboratory, Athens, GA. These viruses were previously passaged once in embryonated chicken eggs. A/New York/55/2004 (H3N2) was kindly provided by Dr. Richard Webby, St. Jude Children's Research Hospital, Memphis, TN. A single stock of these viruses was prepared for use in all assays by inoculating 9-day old specific pathogen-free (SPF) eggs and harvesting the allantoic fluid 48 h post-inoculation. Viral titers were obtained by serial dilution on Madin-Darby canine kidney (MDCK) cells in the presence of 1 µg/ml trypsin (Sigma), and 50% egg infectious doses (EID_50_) were performed in 9-day old SPF chicken embryos and calculated according to the method of Reed and Muench [28].

### Sequencing of influenza hemagglutinin and neuraminidase genes

To determine if mutations in the HA or neuraminidase gene occurred after single egg passage, these genes were sequenced. Briefly, the RNeasy Kit (Qiagen, Valencia, CA) to extract RNA, and the One-step RT-PCR Kit (Qiagen) was employed to amplify the HA and NA gene segments for direct sequencing of PCR products using gene segment-specific amplification primers (Table S1). Full-length amplicons were subjected to purification by agarose gel electrophoresis for cycle sequencing. Cycle sequencing reactions were carried out using an ABI 9700 thermocycler and optimized to produce the maximal length of read while economizing the use of BigDye reagent (Applied Biosystems Inc., Foster City, CA). The resulting 10 µl cycle sequencing reaction was comprised of: 2 µl template, 1 µl ABI BigDye v3.1, 1 µl (1 pmole) sequencing primer, 2 µl ABI 5× sequencing buffer, 4 µl distilled water. Each amplicon was subjected to cycle sequencing reactions using both the forward and reverse amplifying primers. Internal primers were employed to fill in gaps and generate sequence at the 5′ and 3′ termini of each amplicon (Table S2). This scheme resulted in at least two reads for each nucleotide of the sequence. Cycle sequencing reactions were purified using Cleanseq reagent (Agencourt, Beverly, MA) and eluted in 40 µl of 0.1 mM EDTA. Purified cycle sequencing products were loaded onto an ABI 3130XL genetic analyzer and separated by capillary electrophoresis through an 80 cm capillary array. The resulting sequence traces were trimmed and assembled using Sequencher software (Genecodes, Ann Arbor, MI). No mutations in either gene were identified.

### Viral infection of NHBE cells

Human and LPAI viruses were diluted in BEBM (Lonza) to equal titers as determined by MDCK plaque assay. NHBE cells were washed three times with PBS to remove excess mucus secretion on the apical surface prior to infection. Viruses were allowed to adsorb for 1 h at 37°C, the virus dilutions were removed by aspiration and washed again with PBS 3 times. NHBE cells were incubated for the indicated times pi at 37°C. Viruses released apically were harvested by the apical addition and collection of 300 µl of 0.05% BSA-BEBM allowed to equilibrate at 37°C for 30 min. Samples were stored at −80°C until assayed.

### Neuraminidase Treatment and Influenza Infection of NHBE Cells

To remove sia moieties from the cell surface, and to confirm the specificity of lectin binding, NHBE cells were apically treated with the indicated concentration of neuraminidase from *Clostridium perfringens* (Sigma, St. Louis, MO) in PBS for 1 hour at 37°C as previously described [29]. Following sialidase incubation, cells were washed three times with PBS. NHBE cells were apically mock infected or infected with A/Mute Swan/MI/06/451072-2/2006 (H5N1), A/chicken/Pennsylvania/13609/1993 (H5N2), A/chicken/TX/167280-4/02 (H5N3), or NY/04/55/2004 (H3N2) at the indicated multiplicities of infection (MOI). Cells were fixed in 3.7% formaldehyde for 30 min or harvested in triplicate at the times indicated post-infection.

### Quantitative RT-PCR

Total RNA was isolated using RNeasy Mini kit (Qiagen, Valencia, CA) and stored at −80°C until used. Reverse transcription was performed using random hexamers and MuLV reverse transcriptase (Applied Biosystems, Foster City, CA). Influenza M gene expression were measured using a TaqMan real-time quantitative reverse transcriptase PCR (qRT-PCR) assay using previously described primers and probe [30]. Transcript levels were determined following a 10-minute hot start at 95°C in a three-step protocol with 15 s of denaturation (95°C), 30 s of annealing (60°C) and extension at 72°C for 15 s and analyzed using MXPro software by Stratagene (La Jolla, CA). Copy numbers were determined by generation of a standard curve using plasmid DNA encoding influenza M gene. Plasmid DNA concentrations were measured by optical density using a spectrophotometer.

### Flow cytometry analysis of sialic acid residues

NHBE cells staining for sias α2,3 or α2,6 was determined by flow cytometry. Briefly, NHBE cells were washed with PBS and trypsinized for 10 min at 37°C. To determine lectin staining, cells were collected and centrifuged at 220× *g* for 5 min and resuspended in 2% formaldehyde for 30 min on ice and washed with flow buffer (1% BSA, 0.1% NaN_3_ in PBS). To determine the level of sialic acid residues detectable following neuraminidase treatment, trypsinized cells were treated with increasing concentrations of neuraminidase from *Clostridium perfringens* (Sigma, St. Louis, MO) in PBS for 1 hour at 37°C and then fixed in 2% formaldehyde for 30 min on ice and washed with flow buffer. Surface sias expression was determined by primary staining with 20 µg/mL biotinylated *Maackia amurensis* lectin-II (MAA-II) (B-1265, Vector Laboratories, Burlingame, CA) for sias α2,3, or 20 µg/mL biotinylated *Sambucus nigra* lectin (SNA) (B-1305, Vector Laboratories) for sias α2,6 for 1 hour on ice. Secondary staining was performed with APC-conjugated streptavidin (BD, Mountain View, CA) diluted in flow buffer for 1 hour on ice. Cells were washed with flow buffer and analyzed on a LSRII flow cytometer using FACSDiva software (BD). Additional analysis was also performed using FlowJo software (TreeStar, Ashland, OR).

### Confocal Microscopy

NHBE cells were fixed for 30 minutes in 3.7% formaldehyde at the times indicated post-infection. Sialic acid staining was performed as previously described [31]. Briefly, to stain for sias, cells were incubated with 20 µg/mL biotinylated MAA-II (Vector Laboratories) to detect α2,3, or 20 µg/mL biotinylated SNA (Vector Laboratories) to detect sias α2,6 for 1 hour at room temperature, washed with PBS, and incubated with 15 µg/mL Texas Red streptavidin (Vector Laboratories). MAA-II was specifically chosen because it preferentially binds to sias α2-3Galβ1-3(Siaα2-6)GalNAc and not to non-sialic acid residues as do other isoforms of MAA [32]. Following washing, cells were permeabilized in PBS containing 0.5% TX-100, washed in PBS-0.05%TWEEN (PBS-T) and incubated with mouse anti-NP IgG2a diluted in 3% bovine serum albumin (BSA) in PBS-T. The cells were then washed with PBS-T, incubated for one hour with anti-mouse IgG AlexaFluor488 (Molecular Probes, Carlsbad, California) and anti-β-tubulin directly conjugated to FITC (cilia stain). Cells were rapid stained with DAPI (1 µg/mL). After washing with PBS-T, membranes were excised from their culture inserts and mounted on glass slides.

### Glycan array analysis of influenza virus strains

Glycan arrays were used to examine the receptor specificity of the A/Mute Swan/MI/06/451072-2/2006 (H5N1), A/chicken/Pennsylvania/13609/1993 (H5N2), A/chicken/TX/167280-4/02 (H5N3), and A/NY/04/55/2004 (H3N2) strains. Briefly, the strains were purified from allantoic fluid on a 25/60 percent sucrose gradient by ultracentrifugation and resuspended in 1 mM EDTA/PBS. All purified viral stocks were stored at −80°C. Viral titers were determined by standard plaque assay of Madin-Darby kidney cells. The minimum viral titer for glycan analysis was 1×10^5^ pfu/ml, where all viral strains were labeled with 25 µg of AlexaFluor 488 dye (Invitrogen, Carlsbad, CA) in 1 M NaHCO_3_ (pH 9) for 1 hour at 4°C. To remove residual dye, each sample was dialyzed in a 7000 MWCO Slide-A-Lyzer MINI dialysis cassette (Thermo Scientific, Rockford, IL) against 1 mM EDTA/PBS overnight at4°C. The labeled viruses were analyzed via mammalian printed array, version 4.2 (contains 511 glycans) or 5.0 (contains 611 glycans), by the Core H of the Consortium of Functional Glycomics (www.functionalglycomics.org). The PA/93 and MI/06 were evaluated against 511 glycans, while the TX/02 and A/NY were evaluated against 611 glycans. Background fluorescence was determined by averaging the relative fluorescent units (RFU) of all glycans on the array and multiplied by 2. Glycan binding peaks that were above background with a %CV greater than 50% were not considered significant.

### Bead-based detection of cytokines and chemokines

The Luminex® xMAP™ system, a high-throughput microsphere-based suspension array was used with a MILLIPLEX MAP human cytokine/chemokine immunoassay (Millipore, St. Charles, MO) for the rapid immunological detection of secreted cytokines and chemokines from NHBE cell supernatants according to the manufacturer protocol. Briefly, beads coupled with biotinylated anti-IL-1α, anti-IL-1β, anti-IL-8, anti-MCP-1, anti-MIP-1α, anti-MIP1β, anti-IP-10, anti-RANTES monoclonal antibodies were sonicated, mixed, and diluted in bead diluent. For the assay, beads were diluted 1∶4 in bead diluent and incubated overnight at 4°C with NHBE apical wash or basolateral supernatant. After washing, beads were incubated with streptavidin-phycoerythrin for 1 hour at room temperature, washed, and resuspended in wash buffer. The assay was analyzed on a Luminex 200 instrument (Luminex Corporation, Austin, TX) using Luminex xPONENT 3.1 software. Additional analysis was performed using MILLIPLEX Analyst (Millipore).

### Statistical analysis of data

Differences in chemokine expression in Luminex® analysis were evaluated by Student *t* test and considered significant when *p*<0.05. Data are shown as means ± standard deviation (SD).

## Results

### NHBE cells express α2,6 and α2,3 sialic acid receptors

**Figure 1:**
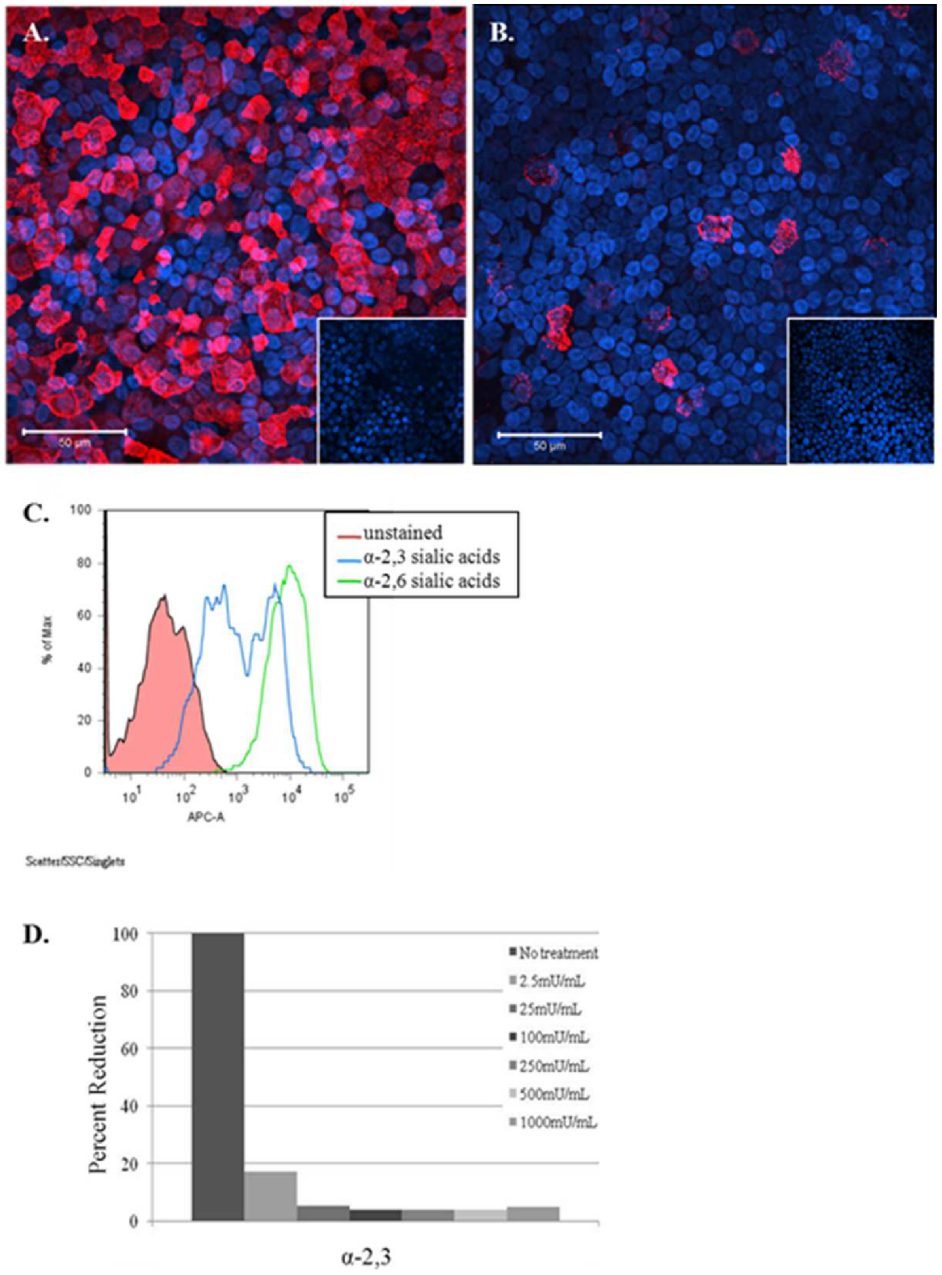
Fully differentiated NHBE cells express both α2,6 and α2,3 sias. NHBE cells were stained for α2,6 (A) or α2,3 (B) sias shown in red. Cells pre-treated with neuraminidase abolished sias residue staining (image inserts). (C) NHBE cells were trypsinized, fixed with 2% formaldehyde, and analyzed by flow cytometry to determine relative percentage of cells staining positive for α2,3 (blue), or α2,6 sia moieties (green). The x-axis shows the mean fluorescence intensity and the y-axis shows the percent positive staining cells. Results shown are representative of four independent experiments. (D) NHBE cells were trypsinized, treated with the indicated concentrations of neuraminidase, and analyzed as in (C) to determine the percentage of cells staining positive for detectable α2,3 sialic acid residues. Results shown are representative of two independent experiments.

**Figure 2:**
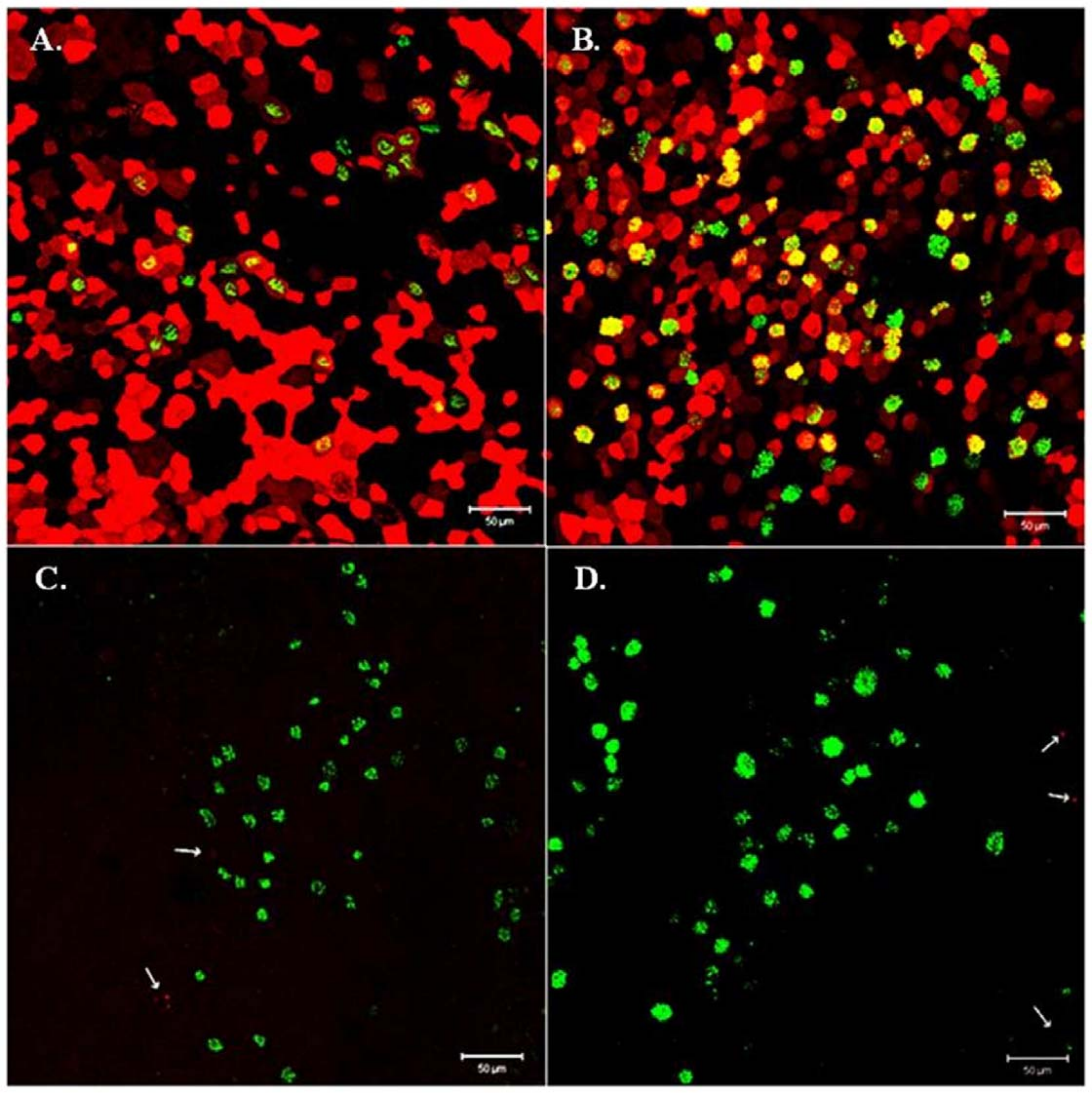
Ciliated and goblet cells express mainly α2,6 sialic acid linkages. NHBE cells were stained for α2,6 (A, B) or α2,3 (C, D) linked sias shown in red, and β-tubulin (A, C) or MUC5AC (B, D) shown in green. Cells expressing a2,3 sias are indicated with arrows. Results shown are representative of two independent experiments.

To determine if the propensity of AIVs to infect NHBE cells was related to sia α2,3 tropism, the cells were stained with sia-specific lectins. MAA-II lectin preferentially binds to α2,3 sialic acids [32], and SNA lectin preferentially binds to α2,6 sialic acids. NHBE cells abundantly express α2,6 sialic acids on the cell surface (Fig. 1A), while α2,3 sias are expressed at a lower level (Fig. 1B). Previous studies suggest that AIV infect ciliated cells which primarily express sias α2,3, while human viruses preferentially infect non-ciliated cells expressing sias α2,6 [24]. The specificity of staining using MAA-II or SNA lectins was confirmed by pre-treating the apical surface of NHBE cells with neuraminidase (image inserts in Figure 2A and 2B) which shows that treatment removed detectable sias from the cell surface.

To determine the relative distribution of α2,3 or α2,6 sias moieties on the NHBE cell surface, the cells were lectin-stained and analyzed by flow cytometry. Figure 1C shows that α2,6 sias are abundantly expressed on most NHBE cells. However, staining for α2,3 sias showed that while many cells express α2,3 sias there are two levels expressed, i.e. dimly positive and brightly positive as determined by flow cytometry. To determine the extent of cell surface sia residues removed by neuraminidase, NHBE cells were treated with increasing levels of neuraminidase (Fig. 1D). NHBE cells treated with the highest neuraminidase concentration (1000 mU/mL) removed >60% all detectable α2,6 sias (data not shown), while similar treatment removed >95% of detectable α2,3 sias.

To determine sias expression on ciliated cells and goblet cells, fully differentiated NHBE cells were immunostained for MUC5AC to indicate goblet cells, and β-tubulin to indicate ciliated cells, and lectin-stained for determining the corresponding surface levels of α2,3 or α2,6 sias. The results show that the NHBE cells have both ciliated and goblet cells (Fig. 2), and while many ciliated and most goblet cells display α2,6 sia residues (Fig. 2A and 2B; co-expression indicated in yellow), none of the ciliated or goblet cells expressed detectable α2,3 sias (Fig. 2C and 2D).

### LPAI virus replicates and are shed from NHBE cells

**Figure 3:**
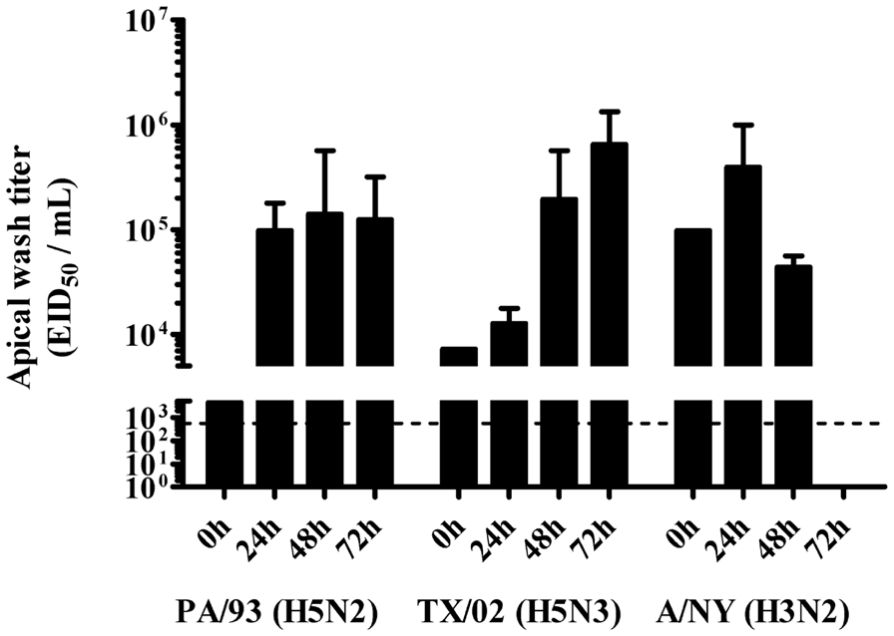
Avian influenza viruses replicate and are shed apically from NHBE cells. NHBE cells were infected with PA/93 (H5N2), TX/02 (H5N3), or A/NY/55/04 at MOI = 0.001. At the times indicated post-infection, BEBM-0.05% BSA was added to the apical surface of the cells and incubated for 30 minutes at 37°C. EID_50_ titers were determined according the method of Reed and Meunch [28]. Data are shown as means ± SD from two independent experiments. The dashed line represents the limit of detection.

To determine if LPAI viruses can infect NHBE cells, the cells were apically infected with A/chicken/Pennsylvania/13609/1993 (H5N2; PA/93), A/chicken/TX/167280-4/02 (H5N3; TX/02), or NY/04/55/04 (H3N2) (Fig. 3) at a multiplicity of infection (MOI) of 0.001 (equivalent to 10^4.38^ EID_50_/mL for PA/93, 10^3.86^ EID_50_/mL for TX/02, or 10^4.99^ EID_50_/mL for NY/04/55/04). This low MOI was chosen to allow for better detection of virus replication in subsequent apical cell washings at the time-points indicated. Within 24 h pi, NHBE cells infected with PA/93 had apical wash virus titers of 10^5^ EID_50_/mL which peaked by 48 h pi to 10^5.8^ EID_50_/mL (Fig. 3). NHBE cells infected with TX/02 had apical wash titers that increased slightly at 24 h pi to 10^4.3^ EID_50_/mL, subsequently increased to 10^5.8^ EID_50_/mL at 48 h pi, and peaked at 10^6.1^ EID_50_/mL at 72 h pi (Fig. 3). As the EID_50_ values were determined from apical washes, the results suggest that both PA/93 and TX/02 replicate and are shed apically from NHBE cells, however we cannot exclude the possibility that virus shed from the basolateral side of the culture did not leak upward toward the apical side. As expected, NHBE cells infected with human influenza NY/04/55/04 (H3N2) supported a productive infection in the first 24 h pi (10^5.9^ EID_50_/mL), but due to considerable cell death related to virus replication, the apical wash titers were decreased by 48 h pi (10^4.8^ EID_50_/mL), and few cells remained at 72 h pi.

### AIVs infect NHBE cells bearing α2,6 sialic acid expression

**Figure 4:**
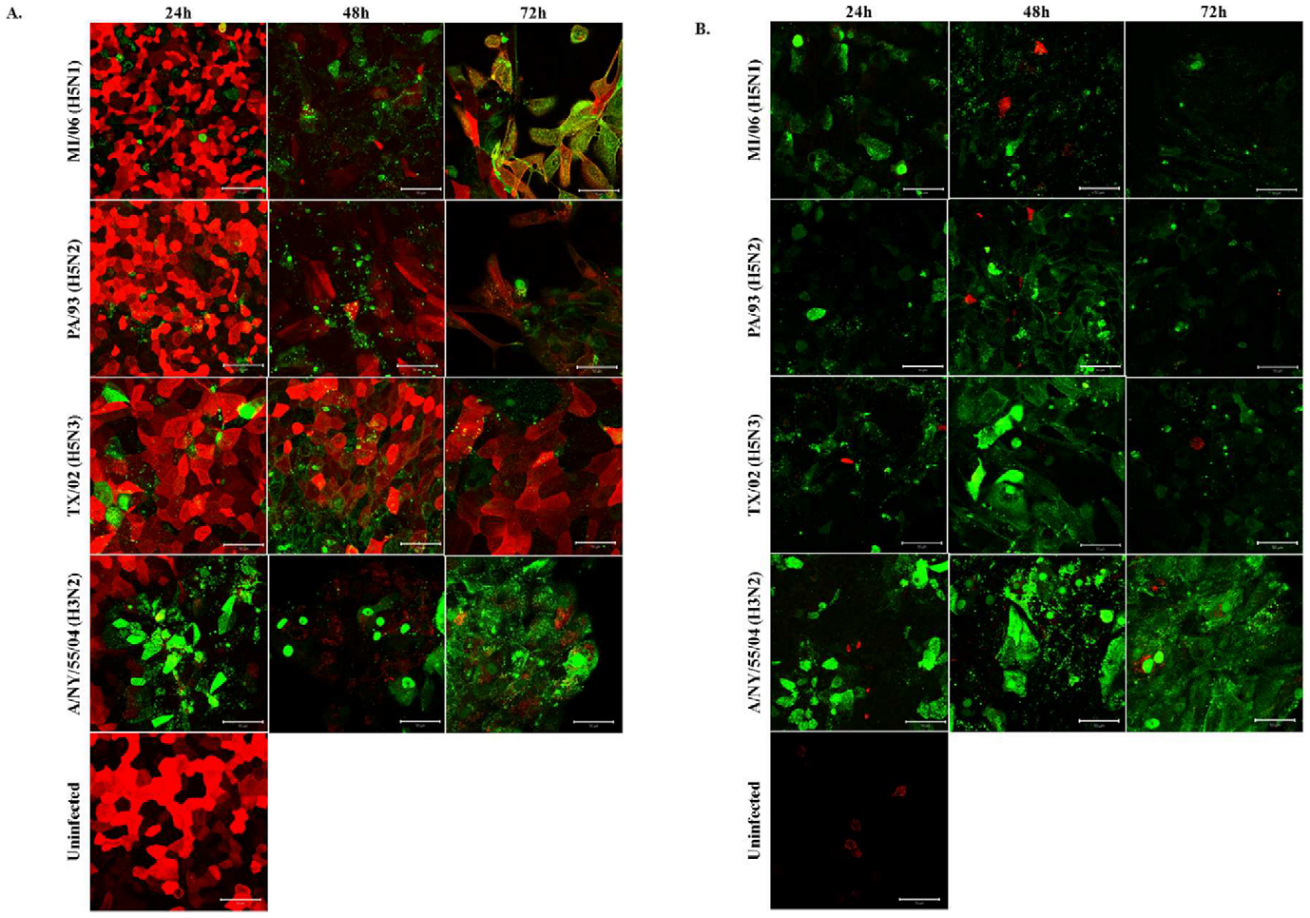
AIVs infect NHBE cells independent of α2,3 sias expression. NHBE cells were infected with MI/06, PA/93, TX/02 or NY/04 at MOI = 0.5. At the times indicated post-infection, cells were fixed with 3.7% formaldehyde in PBS for 30 minutes. Cells were stained for α2,6 (A) or α2,3 (B) sialic acids (red) and influenza NP (green). Results shown are representative of three independent experiments.

To determine if α2,3 sias expression is required for AIV infection of NHBE cells, the cells were infected (MOI = 0.5) with MI/06, PA/93, TX/02, or human A/NY, and lectin-stained for α2,6 sias (Fig. 4A), or α2,3 sias (Fig. 4B), and immunostained for viral NP to detect replicating virus at the time-points indicated. Both human and AIVs infected and replicated in NHBE cells (Fig. 4). The results showing co-staining of α2,6 sias and viral NP at 72 h post-MI/06 infection indicate that this H5N1 wild bird isolate is not restricted to cells expressing α2,3 sias. In addition, the other AIVs infected and replicated in NHBE cells independent of significant α2,3 sias expression (Fig. 4B). For the AIV, replication determined by NP expression, occurred by 24 h and was robust up to 72 h pi where MI/06 (H5N1) and PA/93 (H5N2) replication induced severe cytopathic effects, changes in cell morphology, and loss of the confluent cell monolayer. Similarly, human A/NY (H3N2) quickly spread throughout the NHBE cell culture and induced substantial cytopathic effects and cell loss. Over the time-period of replication by AIVs or human virus, there was a progressive decline of cell surface expression of α2,6 sias, albeit to a lesser extent for TX/02 (H5N3) infection (Fig. 4A). These effects may be linked to influenza neuraminidase expression during replication.

### AIVs infect neuraminidase-treated NHBE cells

**Figure 5:**
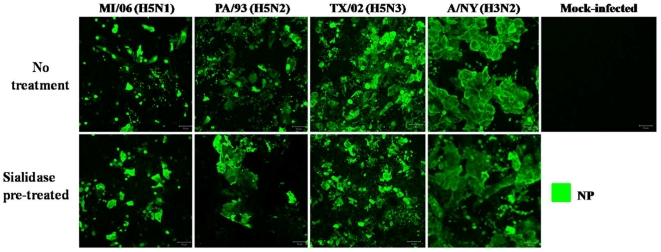
Neuraminidase (sialidase)-treated NHBE cells are robustly infected by AIVs. NHBE cells were mock-treated (top panels) or treated with 25 mU/mL neuraminidase (bottom panels) for 1 hour at 37°C, washed with PBS, and infected with the indicated viruses at a MOI of 0.5. Cells were fixed in 3.7% formaldehyde at 24 h pi and immunostained for influenza NP expression. Results shown are representative of two experiments.

Since AIV infection and replication in NHBE cells did not appear to be constrained by a HA-α2,3 sias barrier (Fig. 5), the NHBE cells were treated with neuraminidase to remove detectable sias and determine if infection could be inhibited. Recent findings suggest that neuraminidase treatment can reduce influenza virus infection, but total inhibition does not occur [33]. Similar to previous findings, neuraminidase treatment of NHBE cells had little effect on AIV or human influenza virus infection, as determined by NP (Fig. 5).

### AIVs predominantly bind α2,3 sialic acid glycans

**Figure 6:**
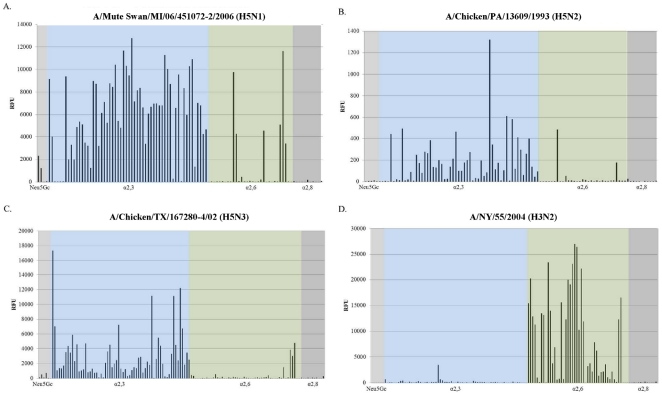
Avian influenza viruses preferentially bind α2,3 glycan moieties. Purified viral stocks (≥10^5^ pfu/ml) labeled with AlexaFluor 488 dye were analyzed via glycan array. The PA/33 and MI/06 were evaluated against 511 glycans, while the TX/02 and NY/04 were evaluated against 611 glycans. The graph represents the N-acetylneuraminic acid (Neu5Ac) and N-glycolylneuraminic acid (Neu5Gc) α2,3, α2,6, and α2,8 glycans. RFU, relative fluorescent units.

Glycan arrays were utilized to further characterize the sialic acid moieties involved in receptor specificity of each virus. The array results show that MI/06, PA/93, and TX/02 avian strains preferentially bind α2,3 glycans with reduced binding observed for α2,6 oligosaccharides (Fig. 6A–C). After closer examination of the α2,6 glycans that appeared to bind MI/06, PA/93, and TX/02 avian strains, several of the glycans were classified as Neu5Aca2-6 glycans because of their terminal position but actually contain α2,3 oligosaccharide branches (e.g. Neu5Aca2-6(Neu5Aca2-3Galb1-3)GalNAca-Sp8) that are presumably utilized in avian specific receptor recognition. Conversely, the human A/NY/55/04 predictably binds the α2,6 sias glycans with less recognition of α2,3 glycans (Fig. 6D). All strains exhibited below background binding to α2,8 glycans (Fig. 6A–D).

### AIVs induce differential chemokine expression patterns by NHBE cells

**Figure 7:**
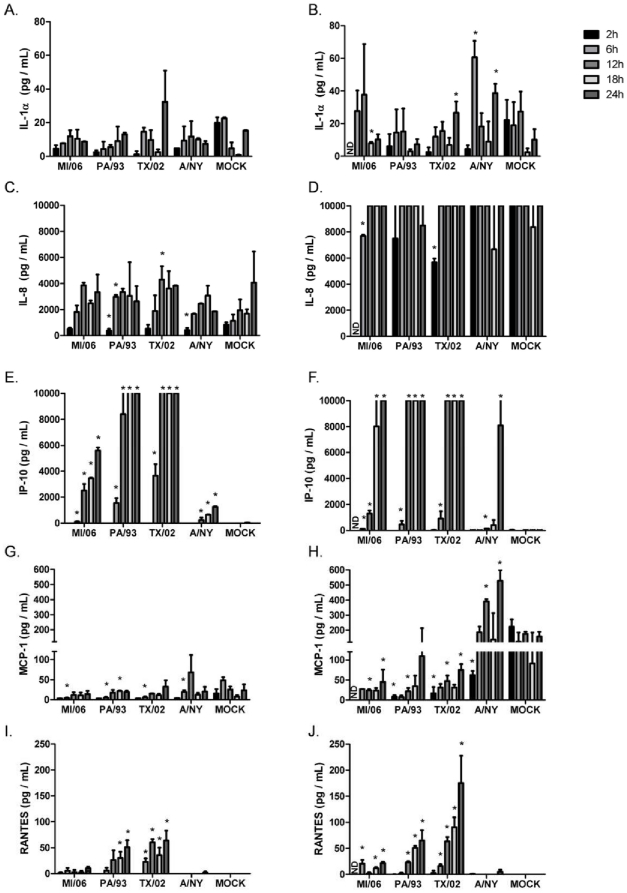
Avian influenza viruses elicit differential chemokine secretion patterns from NHBE cells. NHBE cells were infected in triplicate with the indicated viruses at MOI = 0.5. Apical washes (A, C, E, G, I) and basolateral media (B, D, F, H, J) were collected at the indicated times post-infection and analyzed for the presence of IL-1α (A and B), IL-8 (C and D), MCP-1 (E and F), IP-10 (G and H), and RANTES (I and J). Differences in chemokine expression were evaluated by Student *t* test and considered significant when *p*<0.05. The highest detectable concentration was 10,000 pg/mL. Data are shown as means ± standard deviation (SD). ND, not determined.

Previous studies have shown that pro-inflammatory cytokines and chemokines including interferon (IFN) α/β, interleukin (IL)-1α, IL-1β, IL-6, IL-8, tumor necrosis factor alpha (TNFα), macrophage inflammatory protein (MIP)-1α, MIP-1β, and monocyte chemotactic protein (MCP)-1 are detected at elevated levels in the respiratory tracts of individuals during the acute phase of influenza infection [34], [35]. Patients infected with highly pathogenic H5N1 have been shown to have higher levels of systemic IFNγ, IL-6, interferon-inducible protein of 10 kD (IP-10) and MCP-1 compared to individuals infected with human influenza subtypes [36], [37], [38]. Thus, the NHBE cell model was used to evaluate patterns of chemokine expression induced by human and AIV infection. Both the apical and basolateral secretion patterns of IL-1α, IL-1β, IL-8, IP-10, MIP-1α, MIP-1β, MCP-1, and RANTES expression were determined (Fig. 7). No appreciable levels of IL-1β, MIP-1α or MIP-1β were detected from the basolateral or apical compartments of AIV or human influenza infected NHBE cells (data not shown); however, AIVs induced differential apical and basolateral expression patterns of IL-1a, IL-8, IP-10, MCP-1, and RANTES over time (Fig. 7).

NHBE cells infected (MOI = 0.5) with TX/02 (H5N3) induced higher apical IL-1α expression and significantly more basolateral expression (p<0.05), evident at 24 h pi, compared to infection with the other AIV viruses or mock treatment (Fig. 7A, B). In contrast, MI/06 (H5N1) induced a higher level of basolateral IL-1α expression compared to the other AIV between 2–6 h pi (Fig. 7B). Likewise, human A/NY infection induced a significantly (p<0.05) higher level of basolateral IL-1α expression at 6 h and 24 h pi compared to mock controls (Fig. 7B).

Apically-expressed IL-8 levels detected following AIV or human influenza virus infection were similar, and expressed to slightly higher but insignificant (p<0.05) levels compared to mock-treated cells between 6–18 h pi (Fig. 7C). Interestingly, basolateral expression of IL-8 was beyond the upper limits of detection for the assay system between 6 h and 24 h pi following infection by any virus and in mock-treated cells; however, lower levels of basolateral IL-8 were detected for MI/06 and TX/02 infected cells at 2 h pi (Fig. 7D). These findings are consistent with IL-8 being important in communication between the airway epithelium and the stroma, a feature linked to control of airway remodeling [39].

Apical IP-10 expression was differentially induced by AIV infection. Infection of NHBE cells with PA/93 or TX/02 induced apical IP-10 expression that was low at 6 h pi but significantly (p<0.05) higher than mock treated cells, and between 6 h and 24 h pi, IP-10 levels substantially increased to levels beyond the upper limits of detection of the assay system (Fig. 7E). In contrast, insignificant levels of apical IP-10 expression were detected 2 h post-MI/06 virus infection compared to mock controls, but between 6 h–24 h, apical IP-10 levels steadily increased peaking at 24 h pi. Of note, PA/93 and TX/02 strains were isolated from chickens, while MI/06 is a wild bird (mute swan) isolate. These results suggest that differential levels of IP-10 expression may characterize a unique host response to avian isolates. This feature may also be relevant for unique host responses between AIV and human viruses. NHBE cells infected with A/NY expressed low levels of apical IP-10 relative to AIV infection, although a significant (p<0.05) level of IP-10 expression was evident throughout the time course compared to mock-treated cells. Basolateral expression of IP-10 was similar among AIV. For example, AIVs induced significant (p<0.05) and high IP-10 between 6–24 h pi, while NY/04 infection did not stimulate significant (p<0.05) basolateral IP-10 expression until 24 h pi (Fig. 7F). Similar to IL-8 expression (Fig. 7C and D), avian and human influenza infection of NHBE cells did not induce an appreciable or significant level of apical MCP-1 expression relative to mock-infected cells (Fig. 7G), however, NY/04 infection was associated with an approximate 2-fold significant (p<0.05) increase of basolateral MCP-1 above mock-infected cells at 12 h and 24 h pi (Fig. 7H). Interestingly, infection with AIVs significantly (p<0.05) inhibits basolateral MCP-1 secretion relative to mock-infected NHBE cells. This is in contrast to findings *in vivo* where individuals infected with highly pathogenic H5N1 showed high serum levels of MCP-1 that appeared to correlate with disease severity [36], [38]. It is likely that differences in severity of disease pathogenesis linked to low pathogenic and high pathogenic AIV infections affect MCP-1 expression via differences in levels of inflammation linked to recruitment of different cell types to sites of infection.

Similar to levels of apical IP-10 expression (Fig. 7E), AIV isolated from chickens, i.e. PA/93 (H5N2) and TX/02 (H5N3), induced higher levels of apical and basolateral RANTES expression compared to infection by the wild bird isolate, MI/06 (H5N1) Fig. 7I and J). In addition, AIV also induced higher levels of RANTES expression from both the apical and basolateral surfaces of NHBE cells compared to human A/NY infected cells (Fig. 7I and J). These results are analogous to *in vivo* findings in which individuals infected with highly pathogenic H5N1 had higher systemic levels of RANTES compared to individuals infected with influenza A and B [36]. These results suggest that differential expression of IP-10 (Fig. 7E and F) and RANTES (Fig. 7I and J) during the early response to infection may be a biomarker differentiating AIV from human influenza virus infection, and may highlight host adaptation within avian influenza virus species, i.e. between chicken and wild bird AIV infections.

## Discussion

While AIV primarily infect gastrointestinal epithelial cells of aquatic birds, human influenza viruses primarily infect respiratory epithelial cells. In these studies, we used fully differentiated NHBE cells which closely emulate the human upper respiratory tract epithelium [40]. NHBE cell cultures are recognized as a good *in vitro* correlate to evaluate respiratory virus infection and the host response to infection [41], [42], [43]. HA receptors on human influenza viruses have a preference for cell surface glycans terminating in sias linked to galactose by an α2,6 linkage [11]. Plant lectins have been used to detect α2,3 or α2,6 linkages, which specifically bind to glycolipids and glycoproteins containing sia α2,6 or α2,3 configurations. These sias are expressed on respiratory epithelial cells lining the respiratory tract, e.g. nasal mucosa, trachea, bronchi, bronchioles, and alveoli; however, their abundance varies by tissue location [11] and, at least in culture, by cellular differentiation status [44]. In the tracheal-bronchial tree, human influenza viruses attach predominantly to ciliated epithelial cells [11], [29], [45], [46], but the virus may also attach to non-ciliated cells [24], [29], [47]. At least one explanation for these differences is the MAA preparation used to stain for sias. MAAI and MAAII are both isoforms derived from *Maackia amurensis*, however, MAAI has a greater affinity for SAα2-3Galβ1–4GlcNAc and MAAII has greater affinity for SAα2-3Galβ1–3GalNAc [21], [32], [48], [49]. Binding profiles also showed that MAAI binds to non-sialic acid-containing residues [45].

Influenza A viruses infects a broad range of mammalian species. Interspecies transmission of AIVs, such as human H5N1 infections [50], [51], and the recent swine-origin H1N1 infections [52], [53], [54] have shed light on molecular changes in influenza A viruses that are involved with their adaptation to new species [55]. One recent study suggests that a HA with truncated glycans can recognize α2,3 sias with increased affinity and decreased specificity [56], and single amino acid changes within the HA can lead to complete loss of binding to sias residues and subsequent replication within the lungs [57]. Understanding these features is critical for disease intervention, as these steps are central in emergence of pandemic viruses.

The requirement of HA-sialic acid receptor binding for influenza virus infection has been recognized as a target for disease intervention. Recent studies suggest that an inhaled neuraminidase fusion protein can be used to removal of sias from the airway epithelium as a possible prophylactic and treatment for influenza infection [58]. The rationale for this approach centers on the hypothesis that α2,3- and α2,6 sias on human airway epithelium are in large part barriers for avian and human viruses, and that reducing sias levels on the airway surface would have significant impact on influenza virus infectivity. In this study, we confirmed using NHBE cells that human bronchial epithelial cells express both forms of sialic acid, and that α2,6 sias are more abundant than α2,3 sias. While we show higher levels of sias staining by flow cytometry than by immunofluorescence, this is likely due to the increased sensitivity of flow cytometry as compared to confocal microscopy. We further show that despite neuraminidase treatment, NHBE cells are readily infected by AIV and human influenza strains. These findings are consistent with similar studies demonstrating that H5N1 influenza can replicate within *ex vivo* human respiratory epithelial tissues, despite the lack of sia α2,3 staining [25]. Moreover, neuraminidase-treated MDCK cells can still be infected with influenza [33], and neuraminidase-treated human airway epithelial cells can be infected with a H3N2 virus [29]. ST6Gal I sialyltransferase knockout mice, which lack the enzyme necessary for the attachment of α2,6 sialic acid to N-linked glycoproteins on the cell surface, can be infected with human influenza and produce similar lung virus titers compared to wild-type mice [59]. Therefore, it is likely sias provide a relatively low-affinity interaction for influenza viruses while other potential influenza virus receptors remain to be identified. Furthermore, one study using recombinant HAs showed that several avian HAs exhibited human-like binding profiles to α2,3 sias [60]. The results from our studies show that in the absence of detectable sias moieties on neuraminidase-treated NHBE cells, both human and AIV can readily infect, and that there is evidence that the wild bird isolate (MI/06; H5N1) also infects and replicates in NHBE cells that co-stain for α2,6 sias. It is important to emphasize that neuraminidase treatment reduced >95% of the α2,3 sias expression on the cell surface, and despite this, AIV had the same level of infection in these cells as compared to mock-treated cells. The AIV strains predictably exhibit α2,3 receptor specificity as illustrated in the glycan array with minimal recognition of α2,6 sias glycans, showing that glycan arrays are not a conclusive means for identifying viral receptor binding. The array contains approximately 100 influenza-specific sialic acid targets with only 32 glycans representing the α2,6 sias repertoire, which is a minor representation of all possible α2,6 sias that may be present in nature. The α2,3 moieties included in the array contained complex modifications (i.e. fucosylation, sulfation) that were excluded from the α2,6 glycans, so with the limited number of α2,6 sias on the glycan array it would be difficult to exclude that these avian strains do not bind α2,6 linked sialic acid receptors. A more comprehensive array would need to be employed to fully characterize the receptor specificity of these AIV strains.

The characteristic indications of uncomplicated influenza infection are often nasal obstruction, cough, sore throat, headache, fever, and myalgia which are due to cellular damage at the site of virus replication, and to the cytokines, chemokines, and other inflammatory mediators expressed at the sites of infection [35]. Studies of humans infected with highly pathogenic H5N1 virus who had severe disease showed that these individuals also had high serum levels of IP-10 and monokine-induced by IFNγ (MIG) [36], and H5N1 viruses induced higher levels of TNFα and IP-10 in human macrophages compared to H1N1 viruses [61]. Furthermore, H5N1 virus has been shown to induce IP-10, IFNβ, RANTES and IL-6 mRNA in human primary alveolar type II epithelial and NHBE cells [62]. Interestingly, a recent study showed that viruses with a predilection for sia α2,3 induced higher levels of proinflammatory cytokines than viruses with sia α2,6 binding specificity [63], and studies with Calu-3 cells (derived from human bronchial epithelium) have shown that H5N1 infection results in a weak anti-viral response characterized by little interferon regulatory factor (IRF)-3 nuclear accumulation, reduced IFNβ production and limited interferon stimulated gene (ISG) induction compared to H3N2 infection [31]. In accordance with a recent study that showed robust induction of IP-10, RANTES, and IL-6 production following infection with HPAI H5N1 in alveolar epithelial cells [64], we show in this study that fully differentiated NHBE cells infected with LPAI H5N1, H5N2 and H5N3 induce robust IP-10 and RANTES responses early during infection compared to human H3N2 infection. Moreover, our results show that the origin of the virus isolates e.g. wild bird vs. poultry, or AIV vs. human, differentially affects chemokine expression. NHBE cells infected with H5N2 and H5N3 viruses of chicken origin induced a more potent chemokine response than H5N1 isolated from a mute swan, where for example, apical IP-10 expression was differentially induced by AIV infection. Similarly, NHBE cells infected with A/NY expressed low levels of apical IP-10 relative to AIV infection. Of note, NHBE cells infected with AIV significantly inhibited basolateral MCP-1 secretion relative to mock-infected NHBE cells. Taken together, these findings indicate that human and AIV induce different patterns of chemokine expression following infection of fully differentiated NHBE cells, suggesting that this may contribute to differences in disease pathogenesis between avian and human influenza virus infections in humans.

## Supporting Information

Table S1 **Gene segment amplification primers.**
(DOCX)

Table S2 **Internal sequencing primers.**
(DOCX)
