# Supplementary material for: Avian Influenza Viruses Infect Primary Human Bronchial Epithelial Cells Unconstrained by Sialic Acid α2,3 Residues
Source: PLoS One. 2011 Jun 23;6(6):e21183. doi: 10.1371/journal.pone.0021183 (PMC3121740; doi:10.1371/journal.pone.0021183)
Supplement: Table S1 — Gene segment amplification primers. (DOCX) [file pone.0021183.s001.docx]

| Table S1. Gene Segment Amplification Primers | | |
| --- | --- | --- |
| Gene | Primer | Sequence (5’🡪3’) |
| NA | *N1 and N2 viruses* |  |
|  | Bm-NA-1 | TATTCGTCTCAGGGAGCAAAAGCAGGAGT |
|  | Bm-NA-1413R | ATATCGTCTCGTATTAGTAGAAACAAGGAGTTTTTT |
|  |  |  |
|  | *N3 virus* |  |
|  | Bm-N3-1 | TATTCGTCTCAGGGAGCAAAAGCAGGTGC |
|  | Bm-N3-1420R | ATATCGTCTCGTATTAGTAGAAACAAGGTGCTTTTT |
|  |  |  |
| HA |  |  |
|  | UGAHA-F | AGCAAAAGCAGGGGTCYAWACTATSAAA |
|  | UGAHA-R | AGTAGAAACAAGGGTGTTTTTAATTATAATCTG |
