# Supplementary material for: Avian Influenza Viruses Infect Primary Human Bronchial Epithelial Cells Unconstrained by Sialic Acid α2,3 Residues
Source: PLoS One. 2011 Jun 23;6(6):e21183. doi: 10.1371/journal.pone.0021183 (PMC3121740; doi:10.1371/journal.pone.0021183)
Supplement: Table S2 — Internal sequencing primers. (DOCX) [file pone.0021183.s002.docx]

| Table S2. Internal Sequencing Primers | | |
| --- | --- | --- |
| Gene | Primer | Sequence (5’🡪3’) |
| *H5N1* |  |  |
| NA | MINA-425F | TCAAGGGGCCTTATTGAATG |
|  | MINA-898F | GAGACAACTGGCATGGTTCA |
|  | MINA-770R | CTGGCCATTACTTGGTCCAT |
|  | MINA-1269R | AATCCAACCCCGTCAGTTCT |
|  |  |  |
| HA | MIHA-295F | TGGTCATACATCGTGGAAAAA |
|  | MIHA-815F | GCTCCTGAATATGCGTACAAAA |
|  | MIHA-633R | TGTTTGTTCAGCTGCATCATT |
|  | MIHA-1146R | ATCCACTTCCCTGCTCATTG |
|  |  |  |
| *H5N2* |  |  |
| NA | PANA-405F | GTTGGGTGTTCCGTTTCACT |
|  | PANA-776F | TTTGGGTATCGGGGATAACA |
|  | PANA-790R | GATGGGATGCTTGTTGACAG |
|  |  |  |
| HA | PAHA-283F | GCCTGTGCTATCCAGGAGAC |
|  | PAHA-1251F | GGAAGATGGGTTTTTGGATG |
|  | PAHA-620R | GACACATAAGTGTTCGAGTTCTGG |
|  | PAHA-1099R | ATCCACTTCCCTGCTCATTG |
|  |  |  |
| *H5N3* |  |  |
| NA | TXNA-347F | GGGAGCACTGCTAGGGACTA |
|  | TXNA-876F | GGAAACAGGGTATGTTTGCAG |
|  | TXNA-713R | TCCAGTAAATCCTGTGATCTGC |
|  | TXNA-1197R | AGGGCTGAAAACAGTCCTTG |
|  |  |  |
| HA | TXHA-287F | GTGCTATCCAGGAGGCTTCA |
|  | TXHA-809F | GCAATCATGAAAAGTGAACTGG |
|  | TXHA-1305F | TGGAAAATGAAAGAACTCTGGA |
|  | TXHA-657R | TGACCGGGATTGACCTCTTA |
|  | TXHA-1140R | TCCCATCAATTGCTTTCTGG |
